# Exploiting HIV-1 protease and reverse transcriptase cross-resistance information for improved drug resistance prediction by means of multi-label classification

**DOI:** 10.1186/s13040-016-0089-1

**Published:** 2016-02-29

**Authors:** Mona Riemenschneider, Robin Senge, Ursula Neumann, Eyke Hüllermeier, Dominik Heider

**Affiliations:** Department of Bioinformatics, Straubing Center of Science, Petersgasse 18, Straubing, 94315 Germany; Department of Computer Science, University of Paderborn, Pohlweg 47, Paderborn, 33098 Germany; Wissenschaftszentrum Weihenstephan, Technische Universität München, Alte Akademie 8, Freising, 85354 Germany; University of Applied Science Weihenstephan-Triesdorf, Am Hofgarten 4, Freising, 85354 Germany

**Keywords:** Infectious diseases, Machine learning, Retrovirus, HIV therapy

## Abstract

**Background:**

Antiretroviral therapy is essential for human immunodeficiency virus (HIV) infected patients to inhibit viral replication and therewith to slow progression of disease and prolong a patient’s life. However, the high mutation rate of HIV can lead to a fast adaptation of the virus under drug pressure and thereby to the evolution of resistant variants. In turn, these variants will lead to the failure of antiretroviral treatment. Moreover, these mutations cannot only lead to resistance against single drugs, but also to cross-resistance, i.e., resistance against drugs that have not yet been applied.

**Methods:**

662 protease sequences and 715 reverse transcriptase sequences with complete resistance profiles were analyzed using machine learning techniques, namely binary relevance classifiers, classifier chains, and ensembles of classifier chains.

**Results:**

In our study, we applied multi-label classification models incorporating cross-resistance information to predict drug resistance for two of the major drug classes used in antiretroviral therapy for HIV-1, namely protease inhibitors (PIs) and non-nucleoside reverse transcriptase inhibitors (NNRTIs). By means of multi-label learning, namely classifier chains (CCs) and ensembles of classifier chains (ECCs), we were able to improve overall prediction accuracy for all drugs compared to hitherto applied binary classification models.

**Conclusions:**

The development of fast and precise models to predict drug resistance in HIV-1 is highly important to enable a highly effective personalized therapy. Cross-resistance information can be exploited to improve prediction accuracy of computational drug resistance models.

**Electronic supplementary material:**

The online version of this article (doi:10.1186/s13040-016-0089-1) contains supplementary material, which is available to authorized users.

## Background

According to estimations by the World Health Organization (WHO) around 35 million people are HIV infected in 2013 worldwide. Moreover, 2.1 million individuals were newly infected in 2013. Although antiretroviral therapy has been steadily improved in the last decades, resistance against antiretroviral drugs is still a serious clinical problem. Driving force of drug resistance is the genetic variation of the virus caused by the high mutation rate paired with a fast replication cycle [1].

An HIV-1 therapy typically contains a combination of three or even four active pharmaceutical ingredients from different drug classes, thus inhibiting different steps in the replication cycle of HIV. Classical therapies employ two nucleoside reverse transcriptase inhibitors (NRTIs) combined with one non-nucleoside reverse transcriptase inhibitor (NNRTI) or one protease inhibitor (PI). New drug classes, such as Integrase Inhibitors (INIs), and entry inhibitors, enable alternative therapies when resistance mutations are already present. PIs prevent viral replication by inhibiting the activity of HIV-1 protease, an enzyme used by the viruses to cleave nascent polypeptides into functional proteins. They are designed to have a high affinity to the catalytic center of the HIV protease, thereby hampering its enzymatic activity. NRTIs and NNRTIs inhibit the activity of the reverse transcriptase (RT). NRTIs are nucleoside analogs, and therefore compete for the RT with the natural nucleosides. An incorporation of an NRTI leads to a premature termination of the viral genome replication. In contrast, NNRTIs are non-competitive inhibitors of the RT. They inhibit the movement of protein domains of the RT that is needed to carry out the process of DNA synthesis.

A combination therapy is highly effective in suppressing viral replication, however, the emergence of resistant HIV-1 variants frequently occurs. An important aspect of resistance mutations, namely the occurrence of cross-resistance, has been addressed only recently. Cross-resistance has been frequently found in HIV, leading to resistance not only against a drug from the current treatment, but also to other not yet applied drugs from the same class. These cross-resistance mutations have been described for almost all drug classes, e.g. for PIs, NRTIs, and NNRTIs [2, 3].

In the recent years, machine learning algorithms have improved the development of mathematical models to predict drug resistance, ranging from simple mutation tables over decision trees [4], support vector machines [5], rule-based systems [6] to random forests [7]. In another study, Brandt et al. [8] used multi-label approaches to predict therapy outcome without genotypic information of the virus. Today, the most widely applied tools for resistance prediction are geno2pheno [9] and HIVdb [10]. Geno2pheno applies support vector machines to classify sequences as resistant or susceptible. The HIVdb algorithm uses penalty scores for each mutation within a sequence. The scores are summed up in order to reflect the level of resistance against a certain drug with levels ranging from susceptible to high-level resistance.

However, the use of cross-resistance profiles to improve resistance prediction was hitherto rather neglected and have been only applied in a few studies so far [11, 12]. We were the first to exploit cross-resistance information to improve computational drug resistance prediction by means of multi-label learning [11]. We demonstrated an increased prediction accuracy for six nucleoside analogues by using multi-label classification (MLC) methods, namely classifier chains (CCs) and ensembles of classifier chains (ECCs) in combination with cross-resistance information. In the current study, we applied the MLC methods described in Heider et al. [11] on protease sequences and non-nucleoside reverse transcriptase sequences to investigate whether higher prediction capabilities compared to binary classification could be achieved.

## Methods

### Data

Protein sequences of the HIV-1 protease (PR) and reverse transcriptase (RT) originated from subtype B strains with data for seven PIs (RTV: Ritonavir, IDV: Indinavir, SQV: Saquinavir, NFV: Nelfinavir, APV: Amprenavir, ATV: Atazanavir, LPV: Lopinavir) and three NNRTIs (NVP: Nevirapine, EFV: Efavirenz, DLV: Delavirdine) with IC_50_ ratios were collected from the HIV Drug Resistance Database [13]. The data was separated into susceptible and resistant by drug-specific cutoffs according to Rhee et al. [13]. We removed sequences from the datasets for which no resistance information was available and excluded ATV and LPV from our classification approach, since too many sequences lacked IC_50_ information, resulting in 662 PR sequences and 715 RT sequences with complete resistance profiles. The protein sequences were then encoded and normalized by Interpol [14] with default settings. The sequences can be found in Additional file 1.

### Multi-label classification

In the current study, we used classifier chains (CCs) and ensembles of classifier chains (ECCs) [15] according to Heider et al. [11]. The CC method learns *m* binary classifiers linked along a chain, each time extending the feature space by all previous labels in the chain. Realizing that the order of labels in the chain may influence the performance of the classifier, and that an optimal order is hard to anticipate, Read et al. [15] propose the use of an ensemble of CC classifiers. This approach combines the predictions of different random orders and, moreover, uses a different sample of the training data to train each member of the ensemble. ECCs have been shown to increase prediction performance over CCs by effectively using a simple voting scheme to aggregate predicted relevance sets of the individual chains. For MLC we applied random forests [16] and logistic regression models as base classifiers. Classifiers were evaluated by the F-measure, the classification rate and the AUC (Area Under the receiver operating characteristic Curve) obtained by five-times 10-fold cross-validation. Moreover, we applied permutation tests on the AUC values [17, 18]. The methodological set up of binary and multi-label classification prediction is shown in Additional file 2. The phi coefficient, as well as the variable importance measurements, i.e., the mean decrease in gini impurity, were calculated according to Heider et al. [11].

## Results and discussion

**Fig. 1:**
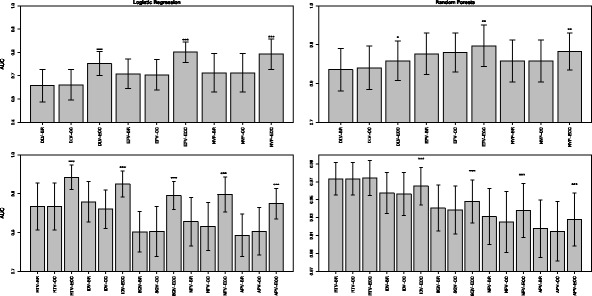
Performance of the different approaches for NNRTIs and PIs. AUC values are shown for NNRTIs (*top*) and PIs (*bottom*), either for logistic regression models (*left*) and random forests (*right*). Significance levels according to Mann-Whitney U test: ***: *p*<0.001; **: *p*<0.01; *: *p*<0.05

Cross-resistance phenomena can be frequently found during antiretroviral therapy and thus have become important targets in research. Our analysis focused on MLC techniques to evaluate the importance of HIV-1 cross-resistance information on drug resistance prediction. Cross-resistance among drugs can be detected by calculating the phi coefficient in a pairwise fashion. The pairwise associations between the labels of all drugs are strongly positive for all PIs as well as for all NNRTIs, with RTV and IDV having the strongest correlation (0.82). For NNRTIs, the strongest association can be observed between NVP and EFV (0.86). Tables 1 and 2 report the phi coeffcients for all PIs and NNRTIs, respectively. The positive correlation between all pairs is further reflected by the results of the variable importance measurements, i.e., the mean decrease in gini impurity of the random forests. A high co-occurrence of sequence peaks can be seen among the drugs in both classes (see Additional files 3 and 4). In NNRTIs mainly three regions show up with significant importance (besides regions with lower importance). Due to the interpolation of sequence length with Interpol, the positions from the importance analyses have to be translated back to sequence positions. Sequence positions 100 and 101 have a high importance for all NNRTIs. For NVP and DLV resistance sequence position 181 seems to be more important than for EFV resistance. Comparing NVP and EFV, also position 190 seems to play an important role in resistance. These findings are in good agreement with known resistance mutations, as positions 100, 101, 181 and 190 are known to be associated with NNRTI resistance in HIV-1. Peaks at multiple sequence positions in the protease sequence can be observed, namely 10, 46, 54, 71, 82, 85 and 90, which are in good agreement with known resistance mutations [19]. Positions 10 and 71 are known to be compensatory, i.e., they compensate for the loss of enzyme activity due to major protease mutations. In order to evaluate the importance of cross-resistance information for drug resistance prediction, we compared three different models: (1) we computed binary models for all labels (one label corresponds to one drug). (2) We constructed CCs by using the label orders according to AUC values of the binary models. (3) We generated ECCs with thirty chains per ensemble with random subset sampling and distinct chain order. The corresponding AUC values of the models are shown in Fig. 1. Results of the other metrics are in accordance with the AUC values (see Additional file 5). All metrics are given as mean +/- sd (standard deviation). The AUC values based on the logistic regression models as well as those based on random forests are significantly higher for ECC compared to BR and CC for all drugs. Moreover, the results of the permutation tests (see Additional file 6) demonstrate the robustness of our models.

**Table 1:** Phi coefficients of NNRTIs

	DLV	EFV	NVP
DLV	1.0000	0.7396	0.7999
EFV	0.7396	1.0000	0.8652
NVP	0.7999	0.8652	1.0000

**Table 2:** Phi coefficients of PIs

	APV	IDV	NFV	RTV	SQV
APV	1.0000	0.6726	0.5921	0.7061	0.6328
IDV	0.6726	1.0000	0.7889	0.8186	0.7040
NFV	0.5921	0.7889	1.0000	0.7465	0.6633
RTV	0.7061	0.8186	0.7465	1.0000	0.7137
SQV	0.6328	0.7040	0.6633	0.7137	1.0000

Taken together, we were able to demonstrate that cross-resistance information can be exploited to improve drug resistance prediction of PIs and NNRTIs by applying MLC techniques, i.e., ECCs. To the best of our knowledge, this is the first time information about NNRTI and PI cross-resistance has been explicitly integrated in HIV-1 drug resistance prediction models. Since we found promising results using MLC methods, the concept could be enhanced in future work by applying alternative MLC methods, including the probabilistic variant of CCs proposed by Dembczynski et al. [20], but also approaches that are not based on the idea of chaining, such as multi-instance learning (MIL) on sequence and structural information to further improve resistance prediction accuracy. A few studies have already reported the use of structural information for drug resistance prediction [21–23], also for data from next-generation-sequencing [24–26]. However, these models neither make use of MIL techniques nor were combined with multi-label approaches yet. Moreover, instead of modeling binary relevance problems, the class membership representation could be expanded to susceptible, intermediate resistance, and resistance, network based approaches [27], or multi-objective optimization [28] could be employed, which might further contribute to refined prediction performance.

## Additional files

Additional file 1 Sequence data. All sequences used in the study with information on cross-resistance. (XLS 402 kb)

Additional file 2 Schematic illustration of the approach. The schematic setting of our MLC approach is shown for PIs. We applied binary classification for each drug using random forests and logistic regression models. The AUC values of binary classification (whereas RTV achieved the best prediction performance, APV the worst) were used to define label order in the CC. For ECCs, ensembles of thirty chains with random order were generated. For training and testing we applied a 10-fold cross-validation scheme. (PDF 20 kb)

Additional file 3 Gini impurity PIs. (PDF 8 kb)

Additional file 4 Gini impurity NNRTIs. (PDF 8 kb)

Additional file 5 Performance measures. (PDF 68 kb)

Additional file 6 Permutation tests. Performance of binary classifier (BR), classifier chains (CCs), and ensembles of classifier chains (ECCs) for each protease inhibitor: The AUC values are shown for real-labeled data and randomized class labels. AUC values are averaged of five runs and shown with standard deviations. light grey: random class labels; dark grey: real-labeled data. (PDF 6 kb)
