# Supplementary material for: Exploiting HIV-1 protease and reverse transcriptase cross-resistance information for improved drug resistance prediction by means of multi-label classification
Source: BioData Min. 2016 Feb 29;9:10. doi: 10.1186/s13040-016-0089-1 (PMC4772363; doi:10.1186/s13040-016-0089-1)
Supplement: Additional file 2 — Schematic illustration of the approach. The schematic setting of our MLC approach is shown for PIs. We applied binary classification for each drug using random forests and logistic regression models. The AUC values of binary classification (whereas RTV achieved the best prediction performance, APV the worst) were used to define label order in the CC. For ECCs, ensembles of thirty chains with random order were generated. For training and testing we applied a 10-fold cross-validation scheme. (PDF 20 kb) [file 13040_2016_89_MOESM2_ESM.pdf]

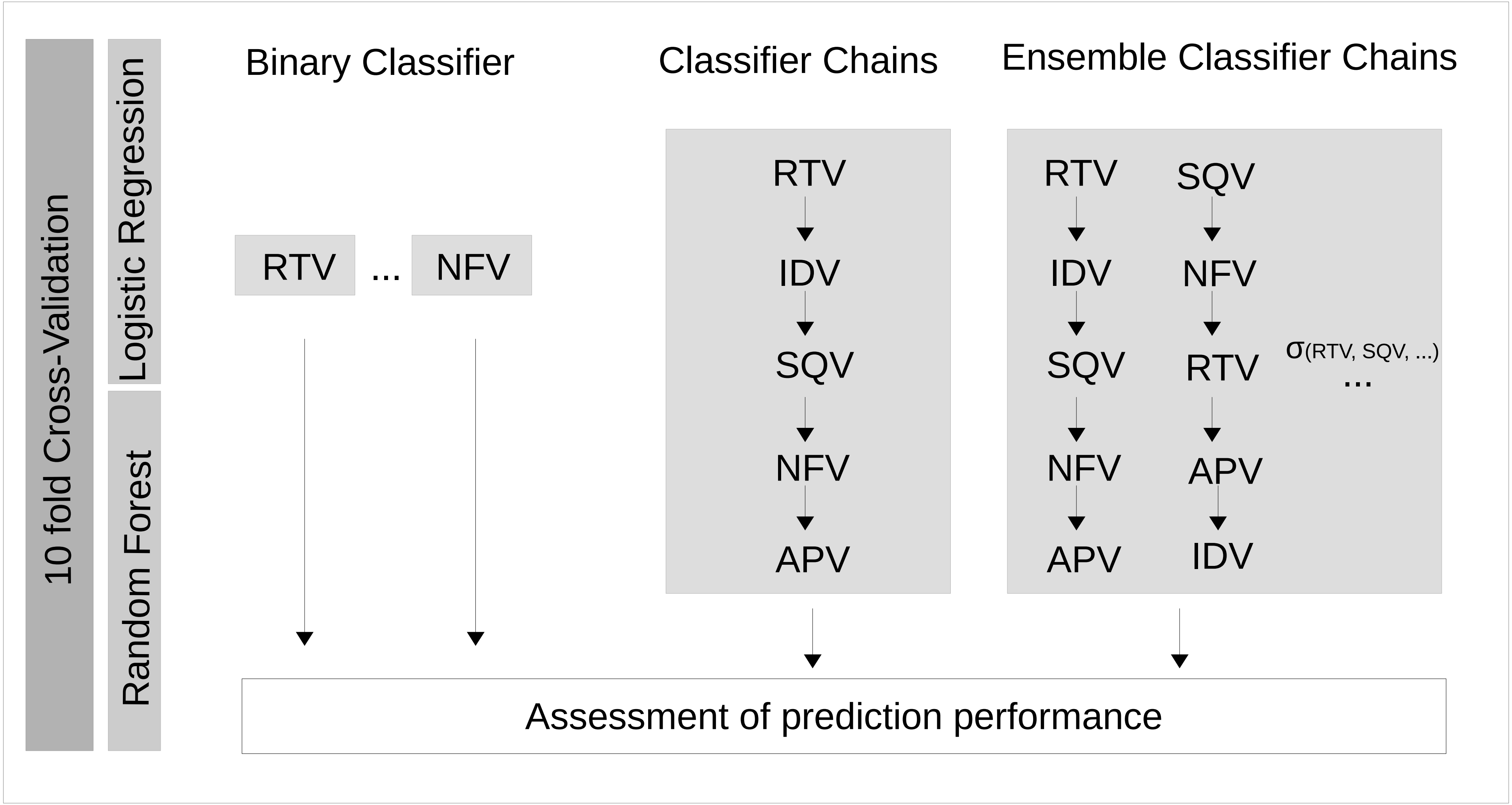
10 fold Cross-Validation
Logistic Regression
Random Forest
Binary Classifier
RTV
...
NFV
Classifier Chains
RTV
IDV
SQV
NFV
APV
Ensemble Classifier Chains
RTV
IDV
SQV
NFV
APV
SQV
NFV
RTV
APV
IDV
σ(RTV, SQV, ...)
...
Assessment of prediction performance
