# Supplementary figures and images for: Exploiting HIV-1 protease and reverse transcriptase cross-resistance information for improved drug resistance prediction by means of multi-label classification
Source: BioData Min. 2016 Feb 29;9:10. doi: 10.1186/s13040-016-0089-1 (PMC4772363; doi:10.1186/s13040-016-0089-1)

**RTV**

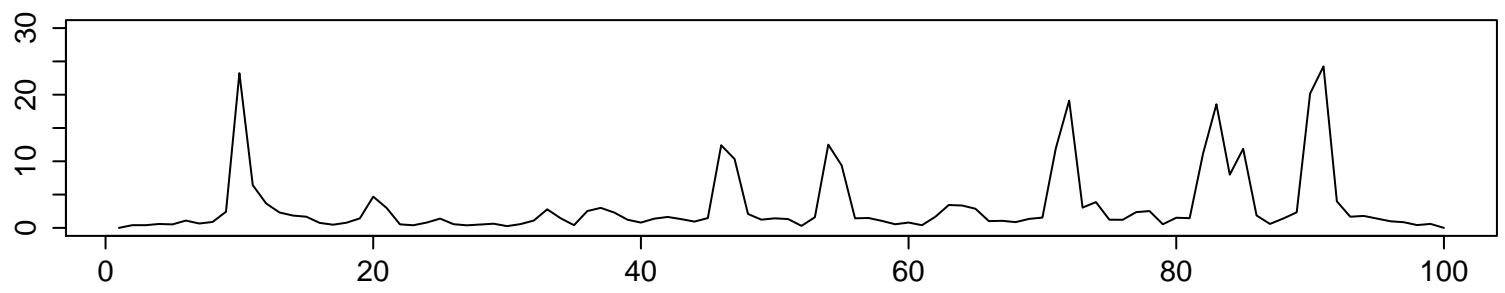

**IDV**

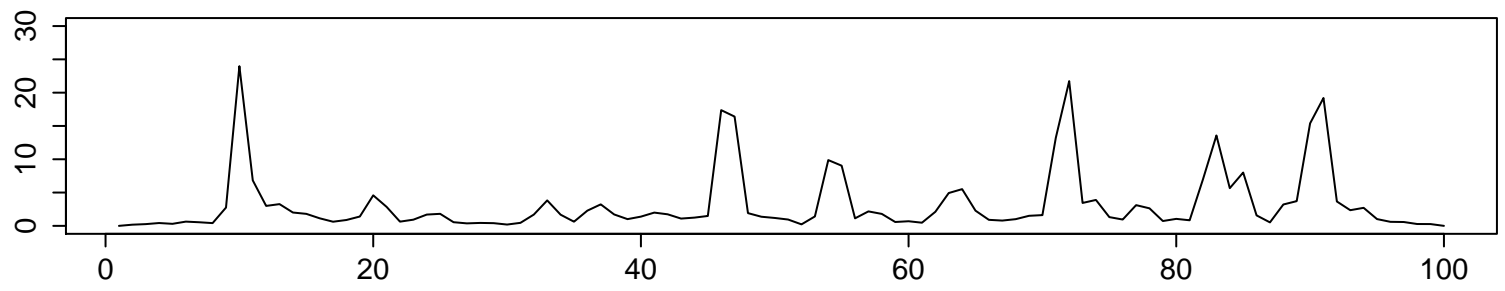

**SQV**

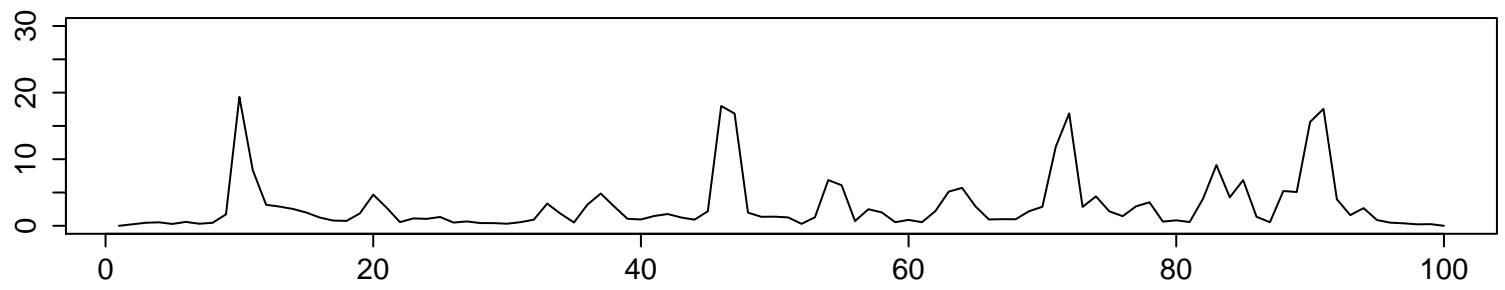

**NFV**

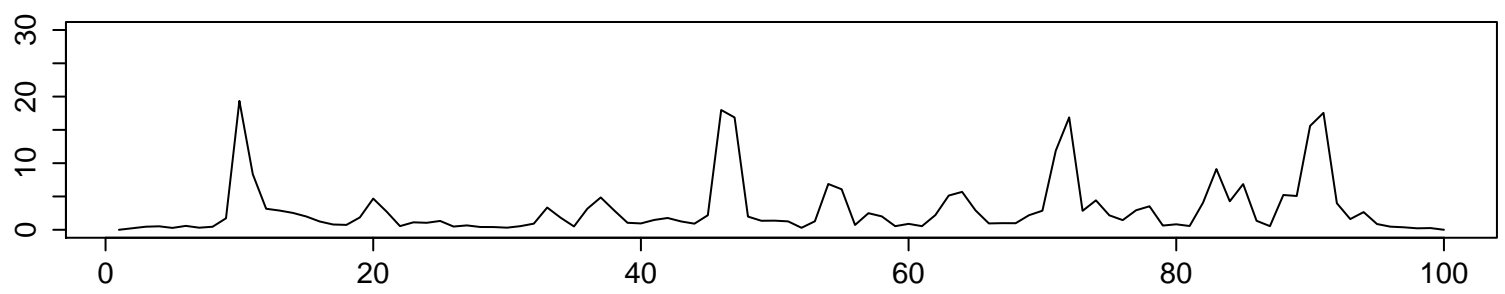

**APV**

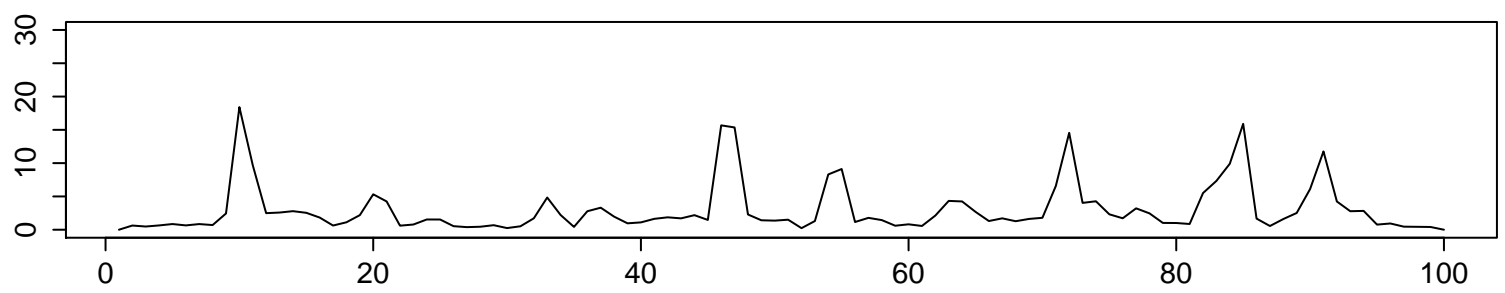

Supplement: Additional file 3 — Gini impurity PIs. (PDF 8 kb) [file 13040_2016_89_MOESM3_ESM.pdf]

**NVP**

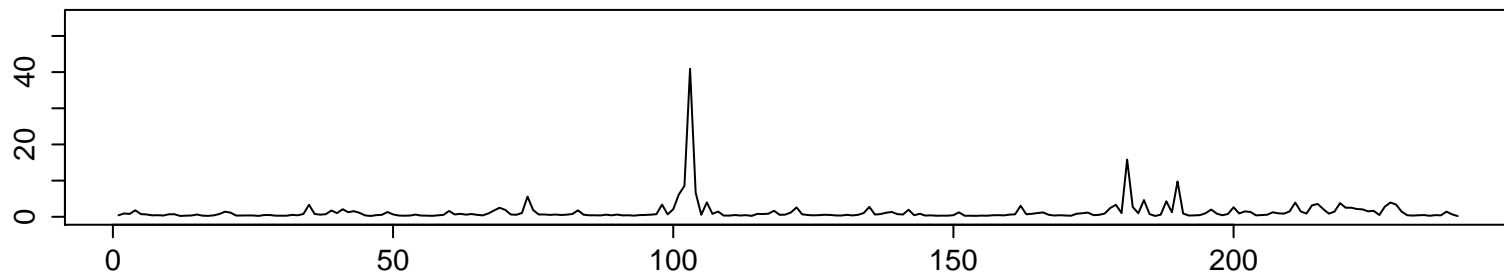

**EFV**

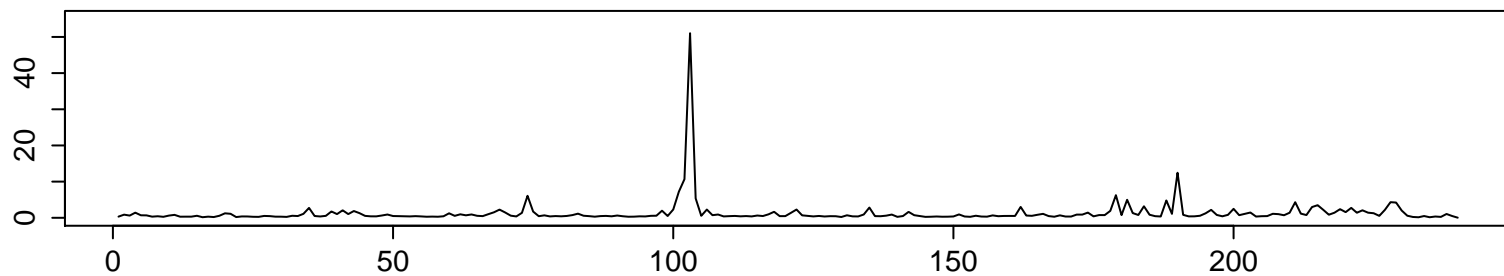

**DLV**

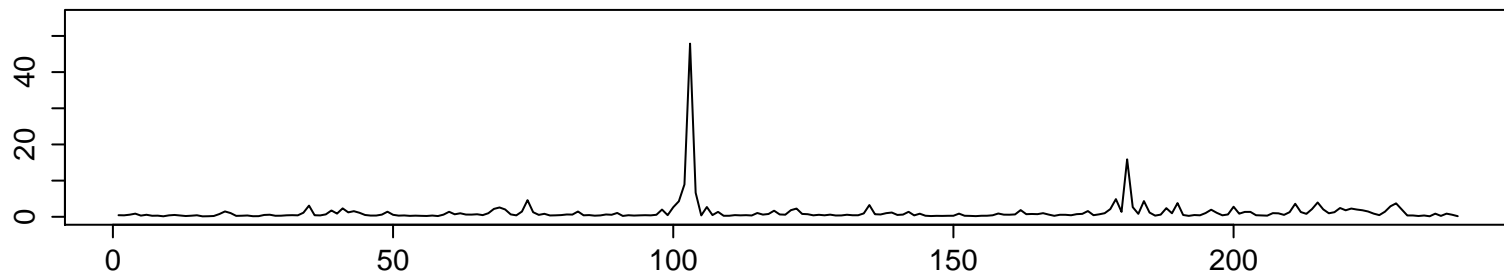

Supplement: Additional file 4 — Gini impurity NNRTIs. (PDF 8 kb) [file 13040_2016_89_MOESM4_ESM.pdf]

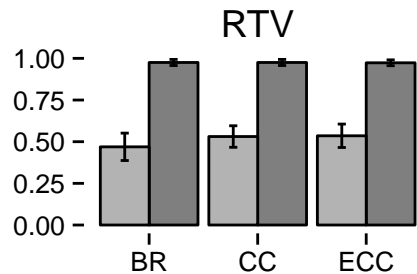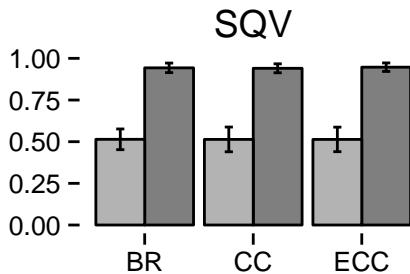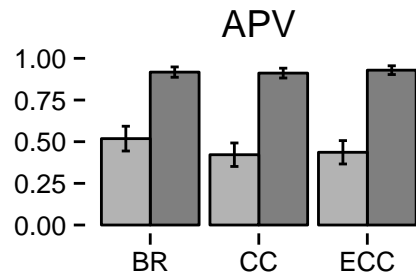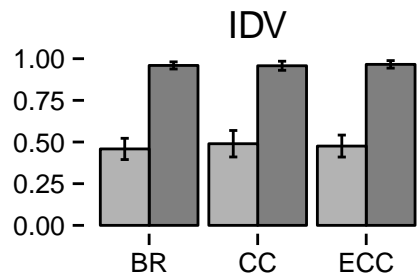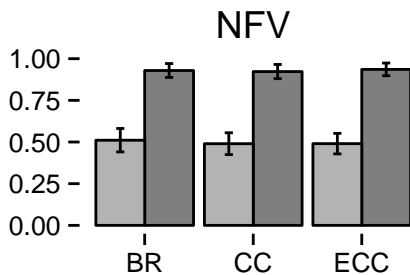

Supplement: Additional file 6 — Permutation tests. Performance of binary classifier (BR), classifier chains (CCs), and ensembles of classifier chains (ECCs) for each protease inhibitor: The AUC values are shown for real-labeled data and randomized class labels. AUC values are averaged of five runs and shown with standard deviations. light grey: random class labels; dark grey: real-labeled data. (PDF 6 kb) [file 13040_2016_89_MOESM6_ESM.pdf]
