# Supplementary material for: Exploiting HIV-1 protease and reverse transcriptase cross-resistance information for improved drug resistance prediction by means of multi-label classification
Source: BioData Min. 2016 Feb 29;9:10. doi: 10.1186/s13040-016-0089-1 (PMC4772363; doi:10.1186/s13040-016-0089-1)
Supplement: Additional file 5 — Performance measures. (PDF 68 kb) [file 13040_2016_89_MOESM5_ESM.pdf]

Exploiting HIV-1 protease and reverse transcriptase cross-resistance information for improved drug resistance prediction by means of multi-label classification

Additional file 4

Mona Riemenschneider, Robin Senge, Ursula Neumann, Eyke Hüllermeier, Dominik Heider

Performance measures

Table 1: Classification performance for PIs: Random forests with 16 trees were used as learning algorithm. Label-wise metrics are shown with mean ± standard deviation in order BR, CC and ECC.

Drugs	Classification rate	AUC	F-measure
RTV	0.9295 ± 0.0284	0.9753 ± 0.0183	0.9340 ± 0.0282
IDV	0.8943 ± 0.0318	0.9595 ± 0.0214	0.8924 ± 0.0341
SQV	0.8629 ± 0.0411	0.9431 ± 0.0287	0.8300 ± 0.0527
NFV	0.8721 ± 0.0431	0.9292 ± 0.0416	0.8923 ± 0.0374
APV	0.8384 ± 0.0383	0.9175 ± 0.0310	0.7798 ± 0.0636
RTV	0.9295 ± 0.0284	0.9753 ± 0.0183	0.9340 ± 0.0282
IDV	0.8983 ± 0.0393	0.9576 ± 0.0272	0.8996 ± 0.0391
SQV	0.8630 ± 0.0385	0.9404 ± 0.0269	0.8351 ± 0.0514
NFV	0.8756 ± 0.0362	0.9228 ± 0.0426	0.8944 ± 0.0335
APV	0.8269 ± 0.0369	0.9115 ± 0.0294	0.7821 ± 0.0546
RTV	0.9421 ± 0.0273	0.9734 ± 0.0176	0.9456 ± 0.0269
IDV	0.9068 ± 0.0386	0.9660 ± 0.0226	0.9077 ± 0.0384
SQV	0.8660 ± 0.0315	0.9470 ± 0.0256	0.8392 ± 0.0428
NFV	0.8877 ± 0.0355	0.9359 ± 0.0382	0.9056 ± 0.0307
APV	0.8449 ± 0.0358	0.9291 ± 0.0263	0.7984 ± 0.0556

Table 2: Classification performance for PIs: Logistic regression was used as learning algorithm. Label-wise metrics are shown with mean ± standard deviation in order BR, CC and ECC.

Drugs	Classification rate	AUC	F-measure
RTV	0.8586 ± 0.0520	0.8669 ± 0.0609	0.8642 ± 0.0551
IDV	0.8515 ± 0.0481	0.8791 ± 0.0524	0.8436 ± 0.0564
SQV	0.8061 ± 0.0448	0.8017 ± 0.0522	0.7504 ± 0.0655
NFV	0.8253 ± 0.0515	0.8277 ± 0.0625	0.8472 ± 0.0491
APV	0.7785 ± 0.0457	0.7925 ± 0.0555	0.6907 ± 0.0662
RTV	0.8586 ± 0.0520	0.8669 ± 0.0609	0.8642 ± 0.0551
IDV	0.8389 ± 0.0472	0.8600 ± 0.5050	0.8308 ± 0.0538
SQV	0.7905 ± 0.0527	0.8026 ± 0.0648	0.7297 ± 0.0756
NFV	0.8133 ± 0.0589	0.8156 ± 0.0626	0.8418 ± 0.0517
APV	0.7849 ± 0.0054	0.8029 ± 0.0617	0.7070 ± 0.0774
RTV	0.8772 ± 0.0464	0.9418 ± 0.0319	0.8819 ± 0.0484
IDV	0.8384 ± 0.0396	0.9249 ± 0.0338	0.8306 ± 0.0466
SQV	0.8126 ± 0.0407	0.8949 ± 0.0364	0.7541 ± 0.0570
NFV	0.8248 ± 0.0534	0.8980 ± 0.0452	0.8505 ± 0.0461
APV	0.8051 ± 0.0432	0.8744 ± 0.0399	0.7246 ± 0.0690

Table 3: Classification performance for NNRTIs: Logistic regression was used as learning algorithm. Label-wise metrics are shown with mean ± standard deviation in order BR, CC and ECC.

Drugs	Classification rate	AUC	F-measure
NVP	0.6973 ± 0.0703	0.7127 ± 0.0843	0.6581 ± 0.0731
EFV	0.7128 ± 0.0560	0.7084 ± 0.0642	0.6324 ± 0.0749
DLV	0.6708 ± 0.0589	0.6573 ± 0.0703	0.5596 ± 0.0753
NVP	0.6973 ± 0.0703	0.7127 ± 0.0843	0.6581 ± 0.0731
EFV	0.6945 ± 0.0611	0.7035 ± 0.0663	0.6031 ± 0.0641
DLV	0.6593 ± 0.0568	0.6609 ± 0.0654	0.5386 ± 0.0848
NVP	0.7264 ± 0.0624	0.7932 ± 0.0662	0.6878 ± 0.0703
EFV	0.7394 ± 0.0461	0.8011 ± 0.0445	0.6525 ± 0.0557
DLV	0.7016 ± 0.0504	0.7520 ± 0.0518	0.5829 ± 0.0828

Table 4: Classification performance for NNRTIs: Random forests with 16 trees were used as learning algorithm. Label-wise metrics are shown with mean ± standard deviation in order BR, CC and ECC.

Drugs	Classification rate	AUC	F-measure
NVP	0.8004 ± 0.0509	0.8583 ± 0.0548	0.7534 ± 0.0626
EFV	0.8172 ± 0.0533	0.8764 ± 0.0536	0.7283 ± 0.0813
DLV	0.7930 ± 0.0519	0.8360 ± 0.0553	0.6846 ± 0.0790
NVP	0.8004 ± 0.0509	0.8583 ± 0.0548	0.7534 ± 0.0626
EFV	0.8238 ± 0.0513	0.8799 ± 0.0506	0.7464 ± 0.776
DLV	0.8043 ± 0.0515	0.8406 ± 0.0567	0.7030 ± 0.0860

Table 5: Instance-wise metrics of logistic regression are given with mean ± standard deviation for NNRTIs.

Type	F-measure	Hamming loss
BR	0.5954 ± 0.0593	0.3063 ± 0.0479
CC	0.6040 ± 0.0567	0.3121 ± 0.0463
ECC	0.6354 ± 0.0461	0.2775 ± 0.0389

Table 6: Instance-wise metrics of random forests are given with mean ± standard deviation for NNRTIs.

Type	F-measure	Hamming loss
BR	0.7430 ± 0.0554	0.1965 ± 0.0455
CC	0.7704 ± 0.0588	0.1912 ± 0.0588
ECC	0.7970 ± 0.0554	0.1723 ± 0.0477

Table 7: Instance-wise metrics of logistic regression are given with mean ± standard deviation for PIs.

Type	F-measure	Hamming loss
BR	0.7551 ± 0.0550	0.1760 ± 0.0335
CC	0.7492 ± 0.0557	0.1828 ± 0.0367
ECC	0.7608 ± 0.0464	0.1684 ± 0.0279

Table 8: Instance-wise metrics of random forests are given with mean ± standard deviation for PIs.

Type	F-measure	Hamming loss
BR	0.8336 ± 0.0373	0.1206 ± 0.0176
CC	0.8431 ± 0.0290	0.1213 ± 0.0216
ECC	0.8556 ± 0.0331	0.1105 ± 0.0183
